# Exploring Factors That Influence Public Engagement of Adoptable Pets on Facebook

**DOI:** 10.3390/ani14223217

**Published:** 2024-11-09

**Authors:** Rachel Morrison, Maria Maust-Mohl, Tracy Ferry

**Affiliations:** 1 Department of Psychology, University of North Carolina at Pembroke, Pembroke, NC 28372, USA; tracyaferry@gmail.com; 2 Department of Psychology, Manhattan University, Riverdale, NY 10471, USA; maria.maustmohl@manhattan.edu

**Keywords:** COVID-19 pandemic, dog, cat, animal shelters, animal adoption

## Abstract

Recent statistics suggest that the percentage of animals adopted from shelters or rescue organizations is lower than expected, especially given the number of animals available for adoption. To help increase adoption rates, animal shelters often advertise adoptable animals on webpages and social media. However, little research has examined what information may be more successful in increasing engagement and interest in the animals to ultimately lead to adoption. This exploratory study examined posts of adoptable animals on Facebook pages of 13 different animal shelters in the US to better understand how post content, such as pictures or videos, type/sex of animal, and the environment the animals are depicted in, may influence public engagement. We also sought to examine if there was a difference between posts in 2019 (pre-pandemic) compared to 2020 (pandemic). The findings suggest that posts on social media can successfully promote animals available for adoption, but the type of animal (dog vs. cat) and environment (e.g., home vs. other) can influence the likes and shares of the posts. In addition, the COVID-19 pandemic and a shift to more online activity may have influenced engagement; posts from Fall 2019 had more likes, while posts in Fall 2020 had more shares. Future research should continue to look for trends in the content of posts, as well as content shared on other social media outlets, to provide further clarification about how these posts influence adoptions.

## Simple Summary

Many animal shelters use social media platforms such as Facebook to advertise animals available for adoption. The current study examined the descriptive content of Facebook posts from 13 US animal shelters to determine whether certain types of information may increase post engagement. Analysis was also focused on comparing differences between posts in 2019 (pre-pandemic) and 2020 (pandemic). The results of the study suggest certain content such as the type of animal and the environment in which they were depicted can influence the likes and shares of posts. Posts in 2019 received more likes, while posts in 2020 received more shares. The findings of this study support the use of social media to increase the engagement of posts depicting adoptable animals, but more research is needed to determine how the content of posts influences adoption rates.

## 1. Introduction

Although 66% of US households have at least one pet [1], the number of animals in shelters remains high. According to the 2023 annual report from Shelter Animals Count [2], 6.5 million cats (51%) and dogs (49%) entered shelters and rescues that year. The percentage of animals adopted from shelters or rescue organizations (54% for cats, 46% for dogs) was lower than expected, especially given the number of animals available for adoption [2]. Shelters and rescue organizations have limited space; thus, they seek to provide temporary housing and support for the animals, making the promotion of adoptable animals a priority.

Animal shelters and rescue organizations often promote adoptable pets using their webpages or online searchable databases such as Petfinder [3]; however, social media platforms (e.g., Facebook and Instagram (as of June 2022, the parent company became Meta), YouTube, TikTok, etc.) have more recently become popular tools. Organizations can benefit from using social media platforms through increased visibility, the creation of an online archive, having the ability to edit or update information, and the formation of online connections or relationships [4]. Increased visibility is particularly important for non-profit organizations as an outlet for communicating their messages more effectively [5] and to a wider audience. These key benefits are likely to increase public engagement of animal shelters’ and rescue organizations’ posts, which may explain the increased popularity of using social media platforms. According to the American Society for the Prevention of Cruelty to Animals® (ASPCA®) [6], 75% of the animal shelters and rescue organizations surveyed reported that social media was crucial for attaining their organization’s goals. Not only was Facebook the most used platform, but 88% of the shelters and rescues reported that Facebook had the greatest impact on increasing adoptions [6]. Around the world, Facebook continues to be followed more than other shelter social media pages and accounts for most adoptions [5].

When promoting adoptable animals on Facebook, organizations use several forms of media, such as pictures, videos, text, and emojis. Research has been ongoing to determine which types of media may have a greater impact on perceptions of adoptable animals, adoption success, and the public engagement of posts. For example, some studies have shown that animals depicted in videos were perceived to have more positive traits (e.g., intelligence, obedience, trainability, playfulness) and were seen as more adoptable [7,8]. These findings suggest videos give potential adopters more information about some aspects of an animal’s personality, but this may depend on the type of animal, as well as other information people are looking for in the posts. Regardless of using a video or photo, the appearance of the animal (more for dogs than cats) has been reported to be one of the most important reasons for adopting a specific animal [9] and for engagement with an animal’s profile [10].

Researchers have also examined the influence of physical appearance (e.g., fur color/length, size, ear position, facial expression, etc.) and other features of the animals (e.g., breed, age, sex, body position/pose, etc.) depicted in the videos and images to see how these details may impact adoption rates or perceived adoptability [8,11,12,13]. There have been conflicting findings regarding whether being depicted with a human, depicted as sitting vs. standing, or being depicted in a cage vs. outdoors is more favorable and leads to quicker adoption [11,12,13]. Due to the number and complexity of characteristics that influence people’s perceptions of the animals, more research is needed to determine how they may be presented to increase potential adoption.

As the use of websites and social media increases for online marketing, public engagement can reveal insights about how viewers interact with the information presented about the animals. Studies have reported that increased engagement (clicks on Petfinder profiles) was linked to shorter stays at the shelter and is influenced by the animal’s fur color (non-black animals had more clicks) or items depicted in the animal images (i.e., toys) [3]. A more recent study manipulated the background settings of photos and found that the dog depicted in the photo (dog ID) was related to link clicking behavior, not the background, suggesting individual characteristics of the animals are more important to viewers [10].

Engagement can be measured on social media by looking at the number of likes and shares a post receives or the number of views a video receives. According to Rose et al. [14], the number of shares significantly predicted the number of likes that a post would receive, and the number of posts on a certain topic (such as health or education) significantly predicted the number of shares. Some researchers utilize netnography, which is an approach to studying online communities, to gain a deeper understanding of the feelings, opinions, and reactions (e.g., comments, emojis/likes, and shares) of those who engage with social media content [5]. Videira et al. [5] reported that participants were more likely to be interested in adoption and engage with posts about animals (e.g., likes, comments, and shares) when they felt more sympathetic towards the animal’s situation and when posts included photos and videos. Using a netnographic analysis, they analyzed Facebook posts from three different months (February, September, and November–December) in 2020 and found that most reactions to posts involved shares or the use of emojis. The thumbs up (like) and heart (love) emojis were more frequently used and notably, there were double the number of posts and increased engagement in February (pre-COVID-19 pandemic) compared to the other months [5].

The social distancing and isolation requirements established during the COVID-19 pandemic appeared to have prompted worldwide interest in pet adoptions during that time. Research has suggested that the relative search volume for dog and cat adoptions peaked in April and May of 2020 [15]. Additionally, during the isolation period of the pandemic, some research reported an increase in adoptions [16] and fostering of animals from shelters [17]; however, variability between shelters has also been reported, with some describing decreased adoptions [18]. The number of dogs and cats that entered the shelter (as strays or relinquished pets) was lower than the same period the prior year, which may help explain the finding that there were less adoptions during the pandemic at some shelters [18]. A recent study suggested that for most shelter volunteers, fostering levels remained the same; however, receiving support from the animal shelter and working from home predicted an increase in fostering dogs during the COVID-19 pandemic [17]. Conflicting findings across shelters suggest that the effects may be more nuanced and warrant further research to better understand the impact of the COVID-19 pandemic on pet adoptions.

Few studies have focused specifically on how the content of social media posts influences engagement and potential adoption success. Thus, the purpose of this exploratory study was to describe the ways in which animal shelters advertise adoptable animals on Facebook and examine whether certain types of content (e.g., style/features of photos and videos, subject/tone of text, type/sex of animal, etc.) influence people’s engagement with the posts on Facebook. We predicted differences in engagement (number of likes and shares) depending on the sex of the animal. We also predicted that posts with dogs would have greater engagement than cat posts, and animals depicted in a cage would have greater engagement. Lastly, we predicted posts from Fall 2020 would have greater engagement than posts from Fall 2019 due to the COVID-19 pandemic and the shift to more online activity.

## 2. Materials and Methods

### 2.1. Selection of Animal Shelters

Using a list of counties in North Carolina, USA, we searched for animal shelters that were affiliated with the American Society for the Prevention of Cruelty to Animals (ASPCA®), the Society for the Prevention of Cruelty to Animals (SPCA), and other independent non-profit humane societies that were active in rescuing and adopting animals and that had social media webpages. Facebook was determined to be the main social media outlet used to advertise animals available for adoption, so our subsequent analysis only focused on Facebook pages. Out of the initial list of shelters, we screened the pages to select those that were managed by the shelter staff and that featured regular posting of specific individual animals up for adoption. We selected posts from September to December 2020 because restrictions due to the lockdown phase of the pandemic were being lifted and people had access to the shelters. Online activity was still high, and many people were working from home at this time. To compare activity in 2019, we selected the same months to investigate differences related to the pandemic. A list of 13 animal shelters with Facebook pages was constructed and researchers were randomly assigned to evaluate different shelters.

### 2.2. Procedure

To account for potential changes in the rates of posting and animal adoptions due to the global COVID-19 pandemic, Facebook posts were evaluated for the same months (September–December) in 2019 (prior to the pandemic) and 2020 (during the pandemic) for each animal shelter. Each post that included dogs and cats available for adoption was analyzed to describe the content of the post including the name of the dog or cat, sex, date/time of the post, number of likes and shares, and whether there were photos, videos, and text (See Table 1). Prior to the independent analysis of the Facebook pages, the researchers standardized codes used to characterize the content in the Facebook posts by reviewing several randomly selected posts as a team to establish a consensus on what information to code and to obtain reliability.

In addition to the main details of each post, the researchers also coded characteristics of the photos or videos of each animal and the text included with the post. If there was more than one animal in the photo and the names were listed, details were included for each. If there was more than one photograph of the animal, the researchers coded each photo separately. The following is a subset of the information that was collected and analyzed for the current manuscript: for each photo, the researchers described the environment (e.g., in a cage, home, other, unknown), accessories (e.g., toys, human presence), color (dogs/cats: black versus other), breed (dogs: purebred, mixed, unknown; cats: domestic short hair, domestic medium hair, domestic long hair, purebred, mixed, unknown), and age information (dogs: puppy (birth–1 year), junior (juvenile/young adult) (1–2 years), adult (2–6 years), senior (7–11 years), and geriatric (12+ years) [19]; cat: kitten (birth–1 year), junior (1–2 years), adult (3–6 years), mature (7–10 years), senior (11–14 years), and geriatric (15+ years). For the environment category, “other” referred to animals that were outside of a cage/run and were in a room or yard at the shelter, in a car, or at an adoption event (e.g., PetSmart, etc.). If any of these categories were not obvious or not stated, the information was listed as “unknown”. Unknown was often used when there was a close-up image of the animal and it could not be determined what environment the animal was in. For the text of the post, the researchers noted if there was use of anthropomorphism (e.g., post written as if the animal was talking), the tone of the post (positive, negative, or neutral), use of emojis, source of the animal (stray, owner surrender, unknown), if it was being fostered (yes or no), and other general comments.

### 2.3. Data Analysis

To examine the general content of the posts, we first ran descriptive statistics for the coded variables to determine any trends in photos or videos depicting dogs versus cats. Some of the categories were represented by only a few individual animals; thus, some categories were combined into broader categories for additional analyses. These included age groups, (unknown, puppy/kitten, junior, adult/mature, senior/geriatric), breed (purebred, mix, other), environment (in a cage, home, other, or unknown), and accessories (e.g., humans or not). Since likes and shares were collected for each post and not individual pictures, we focused our statistical analyses on posts that depicted one animal. Comparisons were made using *t*-tests to examine differences in likes and shares for cats and dogs and for the sex of the animal (male versus female). Ten of the animal shelters had posts from both 2019 and 2020. Using these 10 shelters, we compared the number of likes and shares to examine differences between the years. Further comparisons were made using ANOVAs and Games–Howell post hoc tests to determine if there were differences in likes and shares based on the environment the animals were pictured in. Lastly, a logistic regression was conducted to determine if certain characteristics of the posts predicted adoption success (as noted by an update to specific posts). All statistical analyses were conducted using SPSS (version 28 & 29).

## 3. Results

We analyzed the content (e.g., images, videos, text, number of likes and shares) of 592 Facebooks posts across 13 shelters located in North Carolina from September 2019 to December 2019 (*n* = 309, 52.2%) and September 2020 to December 2020 (*n* = 283, 47.8%). Posts typically depicted one animal available for adoption (*n* = 461, 77.9%); however, some posts (*n* = 131, 22.1%) included multiple animals. Only 10 of the shelters had posts in both 2019 (*n* = 172) and 2020 (*n* = 198); therefore, only posts from these 10 shelters were included when making year comparisons. Typically, Facebook posts were not updated by the shelters; however, 108 (18.2%) posts across the 13 shelters were updated to note that the animal was adopted. Further analyses were not conducted on the adopted subset of the data because we were unable to confirm this information with the individual shelters.

Across all Facebook posts, we analyzed the content of 1041 photos/videos (for a summary of the content, refer to Table 1). To promote available animals, photos (*n* = 922) were typically used for both cats and dogs, while videos (*n* = 145) were used less frequently. Very few senior/geriatric (*n* = 9) animals were available for adoption and very few descriptions utilized a negative tone (*n* = 17) when describing the animals. Very few photos/videos of dogs displayed the animals in a cage or a run (*n* = 26, 5.8%), while 23.1% (*n* = 136) of cats were depicted in a cage. Most of the dog breeds were listed as unknown (*n* = 271, 60.0%), while 148 (32.7%) were listed as mixed, and most of the cat breeds (*n* = 473, 80.31%) were listed as domestic short hair (see Table 1). Only 198 (19.0%) photos or videos depicted humans with the animals and even fewer depicted the animals with toys (cats: *n* = 64, 10.9%; dogs: *n* = 35, 7.7%).

Statistical analyses were conducted using data from posts that depicted only one animal, because Facebook analytics measure engagement via likes and shares by post rather than individual photos. Independent *t*-tests were conducted to compare the number of likes and shares Facebook posts received when promoting available dogs (*n* = 253, 54.8%) versus cats (*n* = 208, 45.1%). Results showed that posts of cats had significantly fewer likes (mean (*M*) = 40.61, standard deviation (*SD*) = 46.50) than posts of dogs (*M* = 87.99, *SD* = 105.16), *t*(361.27) = 6.44, *p* < 0.001, effect size (*d)* = 0.56. Posts of cats also had significantly fewer shares (*M* = 27.12, *SD* = 36.65) than posts of dogs (*M* = 68.00, *SD* = 203.89), *t*(271.68) = 3.13, *p* < 0.001, *d* = 0.27. These results support the prediction that there would be a difference in the level of engagement (number of likes and shares) depending on the type of animal.

For the 10 shelters that had posts from both years, independent *t*-tests were also conducted to compare the mean number of likes and shares between 2019 and 2020. Facebook posts from 2019 had significantly more likes (*M* = 68.14, *SD* = 76.34) than posts from 2020 (*M* = 41.43, *SD* = 39.67), *t*(248.77) = 4.13, *p* < 0.001, *d* = 0.45. However, Facebook posts from 2020 had significantly more shares (*M* = 48.28, *SD* = 64.46) than posts from 2019 (*M* = 32.80, *SD* = 45.67), *t*(354.18) = 2.69, *p* = 0.004, *d* = 0.27. These results support the prediction that there would be a difference in engagement between the years 2019 and 2020.

Independent *t*-tests were also conducted to determine if the sex of the animal (male, *n* = 243, 54.5% versus female, *n* = 203, 45.5%) impacted post engagement (likes and shares). The animals listed as “unknown” sex (*n* = 15) were excluded from the comparison because of low numbers. There was no significant difference in the number of likes received for male animals (*M* = 71.53, *SD* = 88.53) versus female animals (*M* = 60.51, *SD* = 85.41), *t*(444) = 1.33, *p* = 0.18. In addition, there was no significant difference in the number of shares received for male animals (*M* = 45.67, *SD* = 61.15) versus female animals (*M* = 57.00, *SD* = 222.52), *t*(444) = 0.76, *p* = 0.45. These results did not support the prediction that there would be a difference in the level of engagement depending on the sex of the animal depicted in the posts.

**Figure 1:**
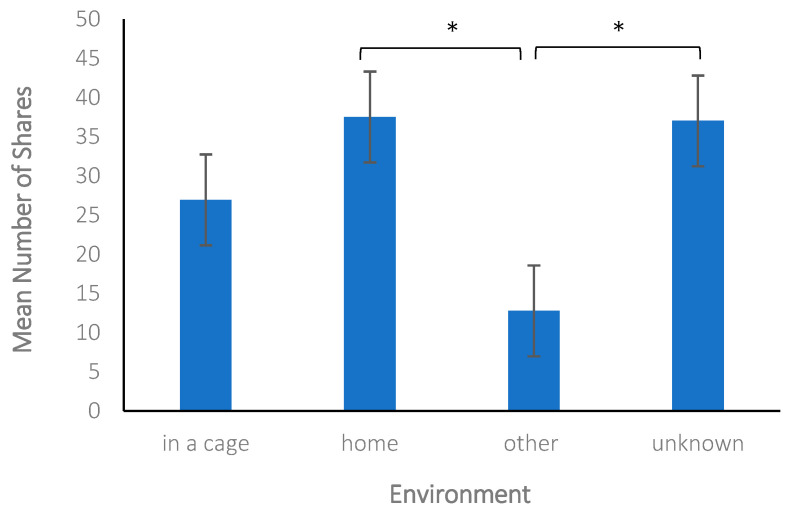
Posts with images of cats in other environments had significantly fewer shares than posts with images of cats in a home and cats in unknown environments (* significant results (*p* < 0.001)).

Additionally, a one-way ANOVA was conducted to determine if the environment (in a cage, *n* = 37, 18.2%; home, *n* = 62, 30.5%; other, *n* = 65, 32.0%; unknown *n* = 39, 19.2%) depicted in the photos of available cats impacted the number of likes and shares posts received. We did not analyze photos of dogs because the environment depicted was rarely categorized as in a cage (*n* = 17, 6.8%); however, there was a trend for those dogs to receive a greater number of mean shares (*M* = 233.41, *SD* = 739.96) and likes (*M* = 181.24, *SD* = 194.48) than dogs in other settings. We found a significant difference in the number of shares a post received based on the environment depicted in the photos of available cats, *F_Welch_*(3, 82.60) = 12.08, *p* < 0.001, η_p_^2^ = 0.09. Games–Howell post hoc tests indicated that posts with images of cats in other environments (*M* = 12.77, *SD* = 16.49) had significantly fewer shares than posts with images of cats in a home (*M* = 37.52, *SD* = 40.10), *p* < 0.001, and cats in unknown environments (*M* = 37.03, *SD* = 30.64), *p* < 0.001 (see Figure 1). We did not find a significant difference in the number of likes a post received based on the environment depicted in the photo, *F_Welch_*(3, 98.89) = 0.18, *p* = 0.91. These results partially support the prediction that there would be a difference in the level of engagement depending on the animal’s environment depicted in the posts.

To determine whether certain characteristics of the posts (species type, sex, year, number of likes, and number of shares) predicted adoption success, as noted by an update to specific posts, we ran a logistic regression analysis. The overall model was statistically significant (χ^2^(5) = 20.45, *p* < 0.001) and explained 6.8% (Nagelkerke R^2^) of the variance in adoptions, which correctly classified 76.5% of the cases. Dogs were 1.71 times more likely to be adopted than cats (*p* = 0.04), but an increased number of likes was associated with a reduction in adoption success (0.995 times, *p* = 0.02) (Table 2).

## 4. Discussion

Animal shelters and other rescue organizations are increasingly using social media tools to promote available animals. The ASPCA® [6] reported that most shelters consider social media an essential tool for attaining their organization’s goals and that Facebook is the most used platform by shelters. In addition, a more recent study reported that the Portuguese Animal Shelter Association’s Facebook page was followed more than their other social media pages, and social media accounted for 95% of the adoptions [5]. In this exploratory study, we characterized the content (text, photos, and videos) of Facebook posts made by animal shelters in North Carolina to promote adoptable dogs and cats, described the impact of this content on engagement, and compared the posting activity of shelters before and during the COVID-19 pandemic. We analyzed 592 posts and summarized the characteristics of the content to identify trends used to advertise adoptable animals (see Table 1). In our examination of posts from 13 animal shelters, we found that videos were used less frequently than photos, which supports prior research by Pyzer et al. [7], who also note that the use of videos has been increasing. Previous studies have shown that videos may help increase the rate of cat and dog adoptions because the animal’s behavioral traits/personality may be perceived more positively (e.g., playful, friendly, intelligent, active) through videos, though this influence is in its early stages of being researched [7,8].

When examining the tone of the posts, most were considered positive or neutral; very few posts used “negative” language (e.g., mention of poor health or sadness) in their descriptions. We expected that shelters may use sad stories to elicit sympathy and empathy in potential adopters, since studies such as that by Videira et al. [5] found that empathy for the animal’s situation was the top factor influencing decisions to adopt. We also expected to see more text that uses anthropomorphism in the descriptions of animals, but we found little use of this language present in the posts. Although the content of the text in each post was not further analyzed, prior studies have shown that clear descriptions of the animal’s behavior in advertisements was preferred and may lead to more successful adoptions [20]. Additional research is needed to confirm what types of content included in the text descriptions influences both engagement and adoptions. Furthermore, few posts examined depicted animals next to a person; therefore, we were unable to determine the impact of a person being present in a photo or video. Past research has suggested that photographing dogs next to a person may increase their perceived adoptable traits, including intelligence or friendliness and decrease perceptions of aggression [21]. However, Isgate and Couchman [11] found that dogs were perceived more favorably when depicted sitting alone versus sitting or walking next to a human. Additional research is necessary to explain these conflicting findings. The presence of toys may also help to influence potential adopters; however, we found that only 99 (9.5%) photos/videos in our analysis depicted animals with toys. Workman and Hoffman [3] found that the presence of toys increased clicks per day for available cats, and Fantuzzi et al. [20] found that cats with toys near them (whether playing with them or not) were viewed more often than cats without toys near them, even while inside a cage. In Lampe and Witte’s [12] study, having a toy present in photos did not appear to influence how quickly dogs were adopted. Although we could not perform a further analysis on the impact of toys, prior research does suggest that shelters may benefit from including toys in their depictions of cats.

Other factors that have been claimed as influential in pet adoption rates and engagement include coat color and age of the animal. Some studies have reported that darker coated animals tend to take longer to get adopted and have less engagement [3]; however, other research indicates a trend in increased positive attitudes towards darker coated animals [22]. Our study was unable to analyze the impact of fur color because very few animals were labeled as black, but future research could explore this by examining the potential connection between animal coat color and adoption influence. In our analysis, nearly half (*n* = 516, 49.6%) of the animals described in the Facebook content were labeled as having an unknown age. Without knowledge of the animal’s history, age determinations beyond broad categories (e.g., puppy/kitten or adult) may not be accurate; thus, we did not examine the influence of age on engagement. Prior research has found that younger animals (i.e., puppies and kittens) are often adopted quicker than older animals [13], and age was reported to be an important reason for why adopters chose their new pet [9]. However, to our knowledge, the impact of age on engagement has not been examined, yet many posts did not indicate the age of the animals, which could be valuable for public interest. In general, these descriptive findings support the idea that information listed or depicted in Facebook posts may influence viewers’ engagement. Therefore, it may be more effective to advertise animals separately and to provide more detail regarding specific characteristics of the animal.

Very few studies have looked specifically at what influences the engagement of animal shelter Facebook posts. The analysis of this study focused on differences in engagement, specifically likes and shares, based on the type of animal promoted and the type of content depicted in the posts. We found cats received significantly fewer likes and fewer shares than posts with dogs, which supported our prediction that there would be a difference based on the type of animal depicted. This finding contrasts the 2023 annual report from Shelter Animals Count [2], which showed that more cats were adopted from shelters than dogs (2.6 million cats, 2.2 million dogs). Although our current research suggests posts of cats may be less popular on Facebook, more research is needed using a more diverse selection of shelters to compare this information across multiple years to better examine this trend. In addition, we found the sex of the animal did not significantly impact the likes or shares of posts, which did not support our initial prediction that there would be a difference. These results support Workman and Hoffman’s [3] study showing there was no difference in the number of clicks animals received per day based on sex. However, they did find that females were typically available for longer. Yet, Nakamura et al. [13] reported that females had shorter stays at the shelter than males, thus demonstrating that further research is needed.

We also examined engagement by comparing the environment in which the animals were depicted, but this analysis was limited to cats because very few dogs were categorized as in a cage or kennel. Notably, even though there were few, there was a trend for posts with dogs depicted in a cage to receive a greater number of mean shares and likes than dogs in other settings. A few individual dogs may have been responsible for this trend; as a result, the influence of depicted environments needs to be investigated further. The environment cats were depicted in appears to be an important factor for shares of posts but not for likes. Specifically, cats that were depicted in “other” environments received fewer shares than those classified in either a home or “unknown”. Many of the images or videos that had environments classified as “unknown” were close-up depictions of the animals. It is possible that animals portrayed this way had more engagement because the viewers were not distracted by environmental features of the images. Prior to the start of the study, we expected animals depicted in cages or in stark conditions would elicit more empathy and concern from viewers [5,13] and thus more engagement online. Although the results were not significant, we found the opposite trend; cats portrayed in cages had the second lowest number of shares and the least number of likes. These results reinforce Workman and Hoffman [3], who reported that profiles with cats photographed outside of cages received more views, suggesting that posts of cats depicted in home environments or close-up depictions may have a more positive impact on viewers.

We also examined if there was a difference in posts between 2019 and 2020 due to increased online activity during the COVID-19 pandemic. The present study found significantly more likes for posts from 2019 and significantly more shares for posts from 2020, supporting our hypothesis but not in a way we had expected. Due to the increased interest in pet adoptions in 2020 [15], we expected that both likes and shares would have been higher in that year. The shift to remote work in 2020 may have motivated people to engage more deeply with social media by commenting and sharing posts to others who may be considering adoption instead of just clicking the like button on a post. However, the subset of data representing updates to posts that certain animals had been adopted suggests that dogs were more likely to be adopted than cats, yet more likes did predict higher adoption success. Since the adoption information was not confirmed with the shelters, these results are presented with caution to suggest more dogs were adopted during this time period, but engagement based on likes and shares may not have been good indicators of public interest. Prior studies have described contradictory findings regarding the impact of the COVID-19 pandemic on pet adoptions [16,18]; thus, future research should continue to examine the trends from this time period and how it has continued to influence the ways that shelters market animals available for adoption.

### Limitations

Although the results of this study identify some meaningful trends in the connection between the content of posts and engagement, there were some limitations to consider. For example, we were not able to confirm with the shelter staff that animals posted on social media were successfully adopted. Posts were occasionally updated with information about whether the pets were adopted or not, but it is still possible the animal may have been returned or an animal may have been adopted outside of the timeframe chosen for analysis. Furthermore, we were unable to determine how long the animals were in the shelter, since the animals may have entered the shelter prior to the posts we viewed and stayed beyond the range of dates we selected. Previous research has suggested that social media can be an impactful resource for promoting animal adoptions; 80% of survey respondents indicated that social media moderately to strongly swayed their decision [3]. However, without confirmation of adoption, it is difficult to determine whether Facebook played a direct role in helping the animals get adopted. In addition, some shelters did not update their posts or did not have detailed information about the animals, limiting the ability to gather data about specifics such as breed or age. Another limitation is the number and inconsistency of posts made between the years. Some shelters had few posts made in a certain year, which limited how much data we could gather for that timeframe. This could have been due to inadequate shelter staffing throughout the COVID-19 pandemic or the irregular use of social media.

## 5. Conclusions

Despite these limitations, the results of this study suggest that animal shelters can influence engagement on their social media pages by being more descriptive in their posts, such as including more detailed information about the dogs and cats and being mindful about the environment the animals are depicted in. To better understand these impacts, future research should examine animal shelters from different geographic locations and different social media outlets. Most importantly, researchers should attempt to gather data directly from shelters that have information available to cross-check internal adoption records with their social media postings. It is clear that the popularity of using Facebook and other social media platforms to promote adoptable animals is not waning; therefore, future research should continue to examine how information presented on these platforms impacts viewers engagement and the likelihood of adopting a pet.

## Figures and Tables

**Table 1:** Summary of Facebook post content for all 13 shelters.

	Dogs		Cats	
	*n*	%	*n*	%
Total Number	452	43.42	589	56.58
Sex				
Unknown	55	12.17	130	22.07
Female	165	36.50	233	39.56
Male	232	51.33	226	38.37
Age				
Unknown	250	55.30	266	45.20
Puppy/Kitten	55	12.20	209	35.50
Junior	43	9.50	38	6.50
Adult/Mature	96	21.20	75	12.70
Senior/Geriatric	8	1.80	1	0.20
Color				
Black	46	10.18	82	13.92
Other	406	89.82	507	86.08
Breed				
Purebred	33	7.30	3	0.50
Mixed	148	32.70	6	1.00
Unknown	271	60.00	29	4.92
Domestic Short Hair	-	-	473	80.31
Domestic Medium Hair	-	-	72	12.30
Domestic Long Hair	-	-	6	1.00
Photo				
Yes	424	93.80	498	84.55
No	28	6.20	91	15.45
Video				
Yes	487	10.40	98	16.60
No	405	89.60	491	83.40
Emojis				
Yes	133	29.40	128	21.70
No	319	70.60	461	78.30
Anthropomorphism				
Yes	62	13.75	68	11.50
No	389	86.25	521	88.50
Tone				
Positive	322	71.40	347	58.90
Neutral	123	27.27	231	39.20
Negative	6	1.33	11	1.90

Note: “*n*” represents the number of animals for each category.

**Table 2:** Logistic regression results for adoption success based on post content.

	B	S.E.	Wald	Sig.	Exp(B)	95% C.I. for EXP(B)	
						Lower	Upper
Dog vs. Cat	0.539	0.256	4.435	0.035	1.714	1.038	2.829
Sex	−0.269	0.230	1.362	0.243	0.764	0.486	1.200
Year	−0.347	0.240	2.078	0.149	0.707	0.441	1.133
Likes	−0.005	0.002	5.689	0.017	0.995	0.990	0.999
Shares	0.003	0.002	3.326	0.068	1.003	1.000	1.007

Note: B = unstandardized regression weight, S.E. = standard error, Wald = the ratio of B value to its S.E. squared, Sig. = *p*-value, and Exp(B) = odds ratio.

## Data Availability

The datasets generated for this study are available upon request to the corresponding author.

## References

[1] Forbes Advisor (2024). Pet Ownership Statistics 2024. https://www.forbes.com/advisor/pet-insurance/pet-ownership-statistics/#sources_section.

[2] Shelter Animals Count (2023). 2023 Statistics. https://www.shelteranimalscount.org/stats.

[3] Workman M.K., Hoffman C.L. (2015). An evaluation of the role the Internet site Petfinder plays in cat adoptions. J. Appl. Anim. Welf. Sci..

[4] Treem J.W., Leonardi P.M. (2013). Social media use in organizations: Exploring the affordances of visibility, editability, persistence, and association. Ann. Int. Commun. Assoc..

[5] Videira M., Nogueira M., Gomes S., Soares A.M., Casais B. (2023). “To adopt or not to adopt, that is the question”: Are social marketing strategies effective to stimulate animal adoption?. Uniting Marketing Efforts for the Common Good-A Challenge for the Fourth Sector. IAPNM 2022. Springer Proceedings in Business and Economics.

[6] American Society for the Prevention of Cruelty to Animals® (2018, September 12). Effectiveness of Social Media Use on Impact of Animal Shelters and Rescue Organizations. https://aspca.app.box.com/s/eu6xvozxrlzytjdhkox9hg0ski6fgklh.

[7] Pyzer C., Clarke L., Montrose V.T. (2017). Effects of video footage versus photographs on perception of dog behavioral traits. J. Appl. Anim. Welf. Sci..

[8] Schoenfeld-Tacher R., Kogan L.R., Carney P.C. (2019). Perception of Cats: Assessing the differences between videos and still pictures on adoptability and associated characteristics. Front. Vet. Sci..

[9] Weiss E., Miller K., Mohan-Gibbons H., Vela C. (2012). Why did you choose this pet?: Adopters and pet selection preferences in five animal shelters in the United States. Animals.

[10] Lamb F., Andrukonis A., Protopopova A. (2021). The role of artificial photo backgrounds of shelter dogs on pet profile clicking and the perception of sociability. PLoS ONE.

[11] Isgate S., Couchman J.J. (2018). What makes a dog adoptable? An eye-tracking investigation. J. Appl. Anim. Welf. Sci..

[12] Lampe R., Witte T.H. (2015). Speed of Dog Adoption: Impact of Online Photo Traits. J. Appl. Anim. Welf. Sci..

[13] Nakamura M., Dhand N., Wilson B.J., Starling M.J., McGreevy P.D. (2020). Picture perfect pups: How do attributes of photographs of dogs in online rescue profiles affect adoption speed?. Animals.

[14] Rose P.E., Hunt K.A., Riley L.M. (2018). Animals in an online world: An evaluation of how zoological collections use social media. J. Zoo Aquar. Res..

[15] Ho J., Hussain S., Sparagano O. (2021). Did the COVID-19 pandemic spark a public interest in pet adoption?. Front. Vet. Sci..

[16] Szydlowski M., Gragg C. (2020). An overview of the current and potential effects of COVID-19 on U.S. animal shelters. AIJR Prepr..

[17] Reeese L.A., Jacobs J., Gembarski J., Opsommer C., Walker B. (2022). The COVID-19 animal fostering boom: Ephemera or chimera?. Animals.

[18] Powell L., Houlihan C., Stone M., Gitlin I., Ji X., Reinhard C.L., Watson B. (2021). Animal shelters’ response to the COVID-19 pandemic: A pilot survey of 14 shelters in the Northeastern United States. Animals.

[19] Harvey N.D. (2021). How old is my dog? Identification of rational age groupings in pet dogs based upon normative age-linked processes. Front. Vet. Sci..

[20] Kelling A.S., Wilson M.L., Martin A.L., Barker S., Mallavarapu S. (2024). Exploring best practices in constructing dog adoption advertisements. Soc. Anim..

[21] Gunter L.M., Barber R.T., Wynne C.D.L. (2016). What’s in a name? Effect of breed perceptions & labeling on attractiveness, adoptions & length of stay for pit-bull type dogs. PLoS ONE.

[22] Fantuzzi J.M., Miller K.A., Weiss E. (2010). Factors relevant to adoption of cats in an animal shelter. J. Appl. Anim. Welf. Sci..

